# Considerations related to vaping as a possible gateway into cigarette smoking: an analytical review

**DOI:** 10.12688/f1000research.16928.3

**Published:** 2019-07-22

**Authors:** Peter N. Lee, Katharine J. Coombs, Esther F. Afolalu

**Affiliations:** 1 P.N.Lee Statistics and Computing Ltd, Sutton, Surrey, SM2 5DA, UK; 2 PMI R&D, Philip Morris Products S.A., Quai JeanRenaud 5, Neuchâtel, CH-2000, Switzerland

**Keywords:** Cigarettes e-cigarettes, gateway effects

## Abstract

**Background: **Compared to cigarette smoking, e-cigarette use is likely to present a reduced risk of smoking-related disease (SRD). However, several studies have shown that vaping predicts smoking initiation and might provide a gateway into smoking for those who otherwise would never have smoked. This paper considers various aspects of the gateway issue in youths.

**Methods: **Here, we reviewed studies (N=15) of the gateway effect examining how extensively they accounted for confounders associated with smoking initiation in youths. We estimated how omitting a confounder, or misclassifying it, might bias the association between vaping and smoking initiation. We assessed how smoking prevalence might be affected by any true gateway effect, and examined trends in youth smoking and e-cigarette use from national surveys.

**Results: **The list of smoking predictors adjusted for in studies reporting a significant gateway effect is not comprehensive, rarely considering internalising/externalising disorders, outcome expectancies, school performance, anxiety, parental smoking and peer attitudes. Furthermore, no study adjusted for residual confounding from inaccurately measured predictors. Better adjustment may substantially reduce the estimated gateway effect. Calculations showed that as any true gateway effects increase, there are much smaller increases in smoking prevalence, and that gateway effects increase only if initiating vaping is more frequent than initiating smoking. These effects on prevalence also depend on the relative odds of quitting vs. initiation. Data from five surveys in US/UK youths all show that, regardless of sex and age, smoking prevalence in 2014–2016 declined faster than predicted by the preceding trend, suggesting the absence of a substantial gateway effect. We also present arguments suggesting that even with some true gateway effect, introducing e-cigarettes likely reduces SRD risk.

**Conclusions: **A true gateway effect in youths has not yet been demonstrated. Even if it were, e-cigarette introduction may well have had a beneficial population health impact.

## Introduction

Recent publications make it clear that, in youths, vaping (i.e., use of e-cigarettes) and cigarette smoking (subsequently referred to as “smoking”) are strongly associated. In the U.S., for example, a survey of ninth and tenth grade students in Hawaii in 2014 ( Wills *et al*., 2017) revealed 195 ever-users of both products, 250 ever-vapers only, 37 ever-smokers only, and 820 never-users of either. From these data, the odds ratio (OR) relating ever-vaping to ever-smoking can be estimated as 17.3 (95% confidence interval (CI) 11.8–25.3). A strong association can also be seen for sixth to twelfth grade students in Texas in 2014 ( Cooper *et al*., 2016), with an OR of 28.8 (25.0–33.1), as well as nationally in 2012 ( Dutra & Glantz, 2014), with an OR of 31.9 (27.6–36.8). Similarly strong relationships are reported also in Canada ( Aleyan *et al*., 2018), France ( Dautzenberg *et al*., 2016), Great Britain ( Eastwood *et al*., 2015), Poland ( Goniewicz *et al*., 2014), and Korea ( Lee *et al*., 2014). Theoretically, this association may arise if vaping encourages smoking, if smoking encourages vaping, and/or if other factors link to use of both products.

Concern that vaping may encourage subsequent smoking, the so-called “gateway” effect, was fuelled by a recent paper by Soneji *et al*. (2017). In this manuscript, the authors combined epidemiological evidence from nine U.S. cohort studies in young people that linked smoking initiation to previous vaping. Among baseline never-smokers, baseline ever-vaping strongly predicted smoking initiation within six to 18 months (OR 3.62, 95% CI 2.42–5.41) after adjusting for various predictors of initiation. Similarly, baseline past 30-day vaping predicted subsequent 30-day cigarette use (OR 4.28, 95% CI 2.52–7.27).

Based on these results and those from one additional study ( Hammond *et al*., 2017), the National Academies Press on the Public Health Consequences of E-Cigarettes ( National Academies of Sciences Engineering and Medicine, 2018) recently concluded that “there is substantial evidence that e-cigarette use increases risk of ever using combustible tobacco cigarettes among youth and young adults, noting that the relevant studies had adjusted for “a wide range of covariates” and considering that it was “unlikely that confounding entirely accounts for the association because reductions in estimates of association from unadjusted to adjusted models were not consistently observed in the literature.”

## Methods

This review considers various methodological aspects of the gateway issue in youths.

### Search parameters

First, based on PubMed searches carried out at intervals starting in May 2017 on “ecigarettes” or “e-cigarettes” or “e-cigs” or “electronic cigarette” or “ecigarette” or “e-cigarette”, we identified papers and reviews that presented results from prospective studies of young people relating to the gateway effect. Further publications were also sought from reference lists of selected papers and reviews. For this review, we also considered studies that provided data on trends over time in cigarette smoking by youths in relation to e-cigarette use, and information on initiation of smoking and e-cigarette smoking by youths relevant to the gateway effect.

A list of factors other than e-cigarette use that are associated with the initiation of cigarette smoking was obtained from US Surgeon General (1994) and from a separate ongoing review on determinants of smoking initiation which aims to present meta-analyses of associations between initiation of cigarette smoking and the various other factors. This identified references using an Embase search in April 2017 with the following search string:

‘smoking’/de OR ‘smoking’ AND (‘initiation’/de OR ‘initiation’) AND (‘prevalence’/de OR ‘prevalence’ OR ‘incidence’/de OR ‘incidence’ OR ‘proportion’ OR ‘age’/de OR ‘age’ OR ‘dual use’ OR ‘combined use’ OR ‘psychosocial’/de OR ‘psychosocial’ OR ‘beliefs’/de OR ‘beliefs’ OR ‘attitudes’/de OR ‘attitudes’ OR ‘perceptions’/de OR ‘perceptions’ OR ‘opinions’ OR ‘acceptance’/de OR ‘acceptance’ OR ‘predictors’/de OR ‘predictors’ OR ‘friend’/de OR ‘friend’ OR ‘family’/de OR ‘family’ OR ‘parent’/de OR ‘parent’ OR ‘propensity score’/de OR ‘propensity score’) AND [humans]/lim AND [english]/lim AND ([embase]/lim OR [medline]/lim)

For the current paper, we only use the results from this search to list those factors shown in one or more studies to strongly associate with initiation of smoking, and to consider the extent to which the identified gateway studies take these factors into account.

### Analysis of search results

In addition, separate sheets of an Excel Program ( Lee, 2019) were developed to:

(a) estimate the effects of omission of a confounding variable, or misclassification of it, under a range of assumptions on the association between vaping and subsequent initiation of smoking, and to
(b) determine, again under various assumptions, how the prevalence of smoking might be affected by any true gateway effect of vaping.

Full details are given in, respectively, Additional Files 2 and 3 ( Lee, 2019). Both sheets are used to provide illustrative examples of how the effects depend on the parameter values assumed.

In the first sheet, the use may determine how the observed gateway effect depends on the following eight parameters:

- The proportion of vapers in never smokers (P _1_)
- The proportion with a particular smoking predictor present in never smokers (P _2_)
- The odds ratio relating vaping to the smoking predictor (K)
- The probability of initiating smoking during follow-up in those who neither smoke nor have the predictor (P _A_)
- The odds ratio for initiating smoking during follow-up associated with vaping - the true gateway effect (G _E_)
- The odds ratio for initiating smoking during follow-up associated with the smoking predictor - the true smoking predictor effect (G _P_)
- The proportion with the predictor who are misclassified as not having it (M _1_), and
- The proportion without the predictor who are misclassified as having it (M _2_)

P _1_, P _2_ and K allow define the baseline population of N never smokers to be subdivided in a 2 × 2 table according to presence or absence of e-cigarettes and of the smoking predictor. P _A_, G _E_ and G _P_ allow determination of the distribution of the population after a single follow-up period. M _1_ and M _2_ are misclassification rates of the predictor.

In the second sheet, the program considers individuals, divided into five equal strata, who initially are all never users of either cigarettes or e-cigarettes. The probability of initiating smoking or vaping increases progressively over the strata, the strata being intended to represent sets of individuals with an increasing susceptibility to tobacco. Over five time intervals, the individuals may transfer to four other groups: current vapers only, current smokers only, current dual users and former users (those who have used one or both products but do not currently use either). Users may modify the values of seven parameters:

- The initiation rate of vaping in the first stratum (P _1_)
- The relative odds of initiation for successive strata (R _1_)
- The relative odds of initiation with smoking compared to vaping (R _2_)
- The relative odds of quitting compared to initiation (R _3_)
- The relative odds of re-initiation compared to initiation (R _4_)
- The relative odds of initiating smoking for vapers compared to tobacco never-users (G _1_)
- The relative odds of quitting smoking for dual users compared to smokers only (G _2_)

Note that R _3_ and R _4_ are each taken to be applicable to either product.

Lastly, the review also includes an analysis of trends in e-cigarette and smoking prevalence in youths based on national surveys which provided annual information over a period of at least 10 years. We identified four surveys from the U.S., the Youth Risk Behavior Survey (YRBS: available from https://www.cdc.gov/healthyyouth/data/yrbs/index.htm), the National Youth Tobacco Survey (NYTS: available from https://www.cdc.gov/tobacco/data_statistics/surveys/nyts/index.htm), the Monitoring the Future study (MTF: available from https://www.icpsr.umich.edu/icpsrweb/NAHDAP/series/35), and the National Survey on Drug Use and Health (NSDUH: available from https://www.samhsa.gov/data/data-we-collect/nsduh-national-survey-drug-use-and-health). We also identified two surveys from the UK, the Smokefree Youth Survey in Great Britain (SYSGB: available from http://ash.org.uk/download/use-of-electronic-cigarettes-among-children-in-great-britain/) and the Office for National Statistics (ONS: available from https://www.ons.gov.uk/peoplepopulationandcommunity/healthandsocialcare/drugusealcoholandsmoking/datasets/ecigaretteuseingreatbritain). All six databases were accessed on 16 Oct 2017.

Based on linear regression of log _e_ (p/(1−p)) on year, where p is the prevalence of smoking, observed prevalences in the years 2014–2016 (a period where the advent of e-cigarettes might have had a measurable effect on smoking prevalence if an important gateway effect existed) was compared with those expected based on the trend in those previous years for which results were available.

## Results

### Evidence relating vaping in youths to subsequent initiation of cigarette smoking

From the literature search, we identified a key systematic review and meta-analysis by Soneji *et al*. (2017) on the association between initial use of e-cigarettes and subsequent cigarette smoking among adolescents and young adult. Table 1 provides details of the nine U.S. studies analyzed by Soneji *et al*. (2017). A total of five studies, conducted with participants with a maximum age of 24–30 years old, collected information using internet-based surveys. The four other studies, involving participants aged less than 20 years old were based on self-completed questionnaires. The follow-up period was 6 months in one study ( Hornik *et al*., 2016), 1 year in five studies ( Leventhal *et al*., 2015; Primack *et al*., 2015; Spindle *et al*., 2017; Unger *et al*., 2016; Wills *et al*., 2017), and 13 to 18 months in the other three studies ( Barrington-Trimis *et al*., 2016; Miech *et al*., 2017; Primack *et al*., 2016). While most of the studies (marked with an A in Table 1) concerned ever- and never-use, two studies (marked with a B) considered current and noncurrent use. While the ORs marked B are less informative as they relate partly to relapse rates, they have been included for completeness.

**Table 1.** Association between vaping at baseline and subsequent smoking among youths.

Source ^a^	Location	Sample size ^b^	Age/grade	Follow-up period (months)	Odds ratio ^c^	Unadjusted OR (95% CI) ^d^	Adjusted OR (95% CI) ^d^
Studies considered by Soneji *et al*. (2017)							
Leventhal *et al*. (2015)	USA, Los Angeles	2558	14	12	A	7.78 (6.15–9.84)	1.75 (1.10–2.78)
Primack *et al*. (2015)	USA, National	694	16–26	12	A	5.66 (1.99–16.07)	8.30 (1.19–58.00)
Barrington-Trimis *et al*. (2016)	USA, Southern California	298	16–18	16	A	5.76 (3.12–10.66)	6.17 (3.29–11.57)
Primack *et al*. (2016)	USA, National	1506	18–30	18	A	6.06 (2.15–17.10)	8.80 (2.37–32.69)
Miech *et al*. (2017)	USA, National	246	17–20	13	A	6.23 (1.57–24.63)	4.78 (1.91–11.96)
Spindle *et al*. (2017)	USA, National	2316	18–25	12	A	3.50 (2.41–5.09)	3.37 (1.91–5.54)
Wills *et al*. (2017)	USA, Hawaii	1141	14–16	12	A	4.25 (2.74–6.61)	2.87 (2.03–4.05)
Hornik *et al*. (2016)	USA, National	1028	13–25	6	B	11.18 (5.41–23.13)	5.43 (2.59–11.58)
Unger *et al*. (2016)	USA, Los Angeles	1056	22–24	12	B	4.71 (2.27–9.77)	3.32 (1.55–7.11)
Other studies							
Best *et al*. (2018)	UK, Scotland	2125	11–18	12	A	4.62 (3.34–6.38)	2.42 (1.63–3.60)
Conner *et al*. (2018)	UK, England	1726	13–14	12	A	5.38 (4.02–7.22)	4.06 (2.94–5.60)
Loukas *et al*. (2018)	USA, Texas	2558	18–25	18	A	2.73 (2.11–3.54)	1.36 (1.01–1.83)
Watkins *et al*. (2018)	USA, National	10384	12–17	12	A	3.50 (2.48–4.94)	2.53 (1.80–3.56)
Hammond *et al*. (2017)	Canada, 2 states	19310	14–18	12	A	4.81 (3.90–5.94)	2.12 (1.68–2.66)
Lozano *et al*. (2017)	Mexico, 3 cities	4695	12–13	20	A	1.82 (1.54–2.14)	1.40 (1.22–1.60)

^a^Two studies ( Hornik *et al*., 2016; Primack *et al*., 2016) were reported only as abstracts, but fuller details were supplied to Soneji *et al*. for their meta-analyses. ^b^Of never- (or noncurrent) smokers at baseline. ^c^A = Odds of smoking initiation, among never-smokers at baseline, for ever- compared with never-vapers at baseline. B = Odds of smoking at baseline, among noncurrent smokers at baseline, for current compared with noncurrent vapers at baseline. ^d^ORs for U.S. studies as given by Soneji *et al*. ORs for U.K. studies come from the source, except that the unadjusted estimates for Best *et al*. and Loukas *et al*. were estimated from data given.

Each study found that baseline vaping significantly predicted subsequent smoking, regardless of covariate adjustment. Adjusted ORs were lower than unadjusted ORs in six studies, particularly in two ( Hornik *et al*., 2016; Leventhal *et al*., 2015). However, two studies ( Primack *et al*., 2015; Primack *et al*., 2016) showed a moderately increased OR after adjustment.

Table 1 also includes results from six later studies cited in a recent review ( Glasser *et al*., 2018): two from the U.S. ( Loukas *et al*., 2018; Watkins *et al*., 2018), two from the U.K. ( Best *et al*., 2018; Conner *et al*., 2018), and one each from Canada ( Hammond *et al*., 2017) and Mexico ( Loukas *et al*., 2018). These results showed an association that reduced but remained significant after adjustment. Two further studies could not be included in Table 1, as they did not provide comparable results. One was a study on young adults in the Chicago area ( Selya *et al*., 2018) that used path analysis and concluded that “E-cigarette use was not significantly associated with later conventional smoking…” The other was a study in the Netherlands ( Treur *et al*., 2018) that reported a strong association after covariate adjustment but did not present unadjusted results for comparison.

There were an additional 15 publications ( Amato *et al*., 2016; Ambrose, 2017; Barrington-Trimis *et al*., 2015; Bold *et al*., 2016; de Lacy *et al*., 2017; Doran *et al*., 2017; Etter & Bullen, 2014; Hanewinkel & Isensee, 2015; Huh & Leventhal, 2016; Kaufman *et al*., 2015; Leventhal *et al*., 2016; Loukas *et al*., 2015; Sutfin *et al*., 2015; Westling *et al*., 2017; Zhong *et al*., 2016) identified in our searches as of possible relevance based on the abstract. However, none of these provided useful data for various reasons. These included studies which: were conducted in adults; predicted e-cigarette use rather than cigarette smoking; did not present results in a form allowing the gateway effect to be estimated; were superseded by later publications; concerned intent to smoke cigarettes and not actual smoking; or even did not consider e-cigarettes at all.

### Association of smoking with other risk factors

Initiation of smoking is long-established to be associated with various sociodemographic, environmental, and behavioral factors. Those identified by an authoritative source over 20 years ago ( US Surgeon General, 1994) include low education level ( Gritz *et al*., 1998; Sargent *et al*., 1997), internalizing/externalizing disorders ( de Leon *et al*., 2002; Ernst *et al*., 2010; Rohde *et al*., 2003), outcome expectancies ( Barrington-Trimis *et al*., 2015; Simons-Morton *et al*., 1999), susceptibility to smoking ( Huang *et al*., 2005; Jackson, 1998), conduct problems ( Dalton *et al*., 2003; Scal *et al*., 2003), substance use ( Reed *et al*., 2007; Scal *et al*., 2003), risk-taking behaviour ( Coogan *et al*., 1998; Dalton *et al*., 2003), poor school performance ( O'Connor *et al*., 2003; Sargent *et al*., 1997), anxiety ( Coogan *et al*., 1998; Scal *et al*., 2003), household smoking ( Picotte *et al*., 2006; Sargent *et al*., 1997), peer smoking ( Barrington-Trimis *et al*., 2015; Picotte *et al*., 2006), peer attitudes to smoking ( Barrington-Trimis *et al*., 2015; Daly *et al*., 1993), low self-esteem ( Dalton *et al*., 2003; Weiss *et al*., 2006), and other tobacco product use ( Barrington-Trimis *et al*., 2016; Jordan *et al*., 2014). Currently available evidence reveals that many of these predictors consistently show a strong association with initiation, with reported ORs often exceeding 10. While these predictors are clearly not all independent, their number illustrates the difficulty in ensuring that gateway studies consider an adequate list. To avoid unfeasibly long questionnaires or huge studies, further work is needed to define an agreed minimum list of factors.

For the 15 studies included in Table 1, Additional File 1 ( Lee, 2019) describes in detail how these risk factors were taken into account. While standard demographics were generally considered, and smoking susceptibility, substance use, risk-taking behaviour, other tobacco use, parental education, family smoking, and peer smoking were considered in at least five studies, many other relevant factors were rarely considered. These include internalizing/externalizing disorders, outcome expectancies, school performance, anxiety, parental smoking, and peer attitudes to smoking. The studies vary in the number of factors accounted for, some ( Conner *et al*., 2018; Leventhal *et al*., 2015; Watkins *et al*., 2018; Wills *et al*., 2017) considering more than 10 factors, others ( Miech *et al*., 2017; Unger *et al*., 2016) only five. As shown in Additional File 1 ( Lee, 2019), the questions used to assess these factors also varied considerably between studies.

Unfortunately, no study reported which factors contributed most to their adjustment. Thus, for example, Leventhal *et al*. (2015) adjusted for the most factors and found that the unadjusted OR of 7.78 (6.15–9.84) reduced dramatically after adjustment to 1.75 (1.10–2.78); however, the authors did not clarify which factors mainly contributed to this reduction.

While the search terms we considered may not have been fully comprehensive, it is clear from the material considered that a wide range of risk factors are related to initiation of smoking and that many of them were considered rarely, if at all, in the gateway studies.

### Bias in the estimated gateway effect due to failure to adjust for a relevant covariate – simple confounding

What effect might failure to adjust for a relevant predictor of smoking have on the estimated gateway effect observed between vaping and smoking? To attempt to gain insight into this question, we consider (using the first sheet of the Excel program we developed) a hypothetical baseline population of N never-smokers, of which P _1_ vape, and P _2_ have the smoking predictor. P _1_ and P _2_ are correlated, with an odds ratio of K. We assume that, during follow up, those who neither have smoked nor have the predictor have a probability of initiating smoking of P _A_ and that the odds of smoking initiation are independently increased by G _E_ for vapers and by G _P_ in those with the smoking predictor.

The illustrative results in Table 2, supported by mathematical detail in Additional File 2 ( Lee, 2019), demonstrate the problem. In the basic Situation 1, we set P _1_ = 0.2, P _2_ = 0.2, K = 5, P _A_ = 0.05, G _E_ = 1, and G _P_ = 4. Instead of observing the true gateway effect (G _E_) of 1, we observe a spurious gateway effect of 1.633 due to the uncontrolled confounding. In Situations 2–6, G _E_ remains at 1, but other parameters are varied. There is a clear tendency for the confounding effect to increase with increasing K (Situation 2) and G _P_ (Situation 3), but varying P _A_ (Situation 4), P _1_ (Situation 5), or P _2_ (Situation 6) has less effect. Increasing G _E_, with the other parameters fixed (Situation 7), increases the observed gateway effect, but the ratio of the observed to the true gateway effect is somewhat reduced, from 1.633 for G _E_ = 1 to 1.352 for G _E_ = 10.

**Table 2.** Bias in the gateway effect due to failure to adjust for the smoking predictor.

Situation	Proportion of vapers in never- smokers (P _1_)	Proportion with predictor in never- smokers (P _2_)	Odds ratio relating vaping and predictor (K)	Probability of smoking in non-vapers where the predictor is absent (P _A_)	True OR of smoking for predictor (G _P_)	True gateway effect (G _E_)	Observed gateway effect
1 – basic	0.2	0.2	5	0.05	4	1	1.633
2 (a)	0.2	0.2	2	0.05	4	1	1.233
(b)			3		4	1	1.399
(c)			10		4	1	1.980
3 (a)	0.2	0.2	5	0.05	2	1	1.266
(b)					7	1	1.983
(c)					10	1	2.218
4 (a)	0.2	0.2	5	0.02	4	1	1.644
(b)				0.1	4	1	1.614
(c)				0.2	4	1	1.573
5 (a)	0.1	0.2	5	0.05	4	1	1.658
(b)	0.3				4	1	1.599
(c)	0.5				4	1	1.520
6 (a)	0.2	0.1	5	0.05	4	1	1.438
(b)		0.3			4	1	1.666
(c)		0.5			4	1	1.510
7 (a)	0.2	0.2	5	0.05	4	2	3.149
(b)					4	4	5.956
(c)					4	10	13.522

### Bias in the estimated gateway effect due to adjustment for an inaccurately measured covariate – residual confounding

While confounding is well understood by epidemiologists, and is the reason why the authors of the gateway papers made some adjustments, “residual confounding,” arising from inaccurately determining confounding variables, is rarely considered. Many statisticians have highlighted this problem of residual confounding. Almost 40 years ago, Greenland (1980) noted that “misclassification of a confounder” leads to “partial loss of ability to control confounding,” while Tzonou *et al*. (1986) later noted that “even misclassification rates as low as 10% can prevent adequate control of confounding.” Other publications ( Ahlbom & Steineck, 1992; Fewell *et al*., 2007; Greenland & Robins, 1985; Phillips & Smith, 1994; Savitz & Barón, 1989) show that if X is an inaccurately measured true cause of disease, and Y, precisely measured, is not a cause but is correlated with X, one may conclude incorrectly that Y, not X, is the cause. None of the gateway papers cited in Table 1, nor Soneji *et al*. (2017), made attempts to adjust for bias from residual confounding, although all except two ( Primack *et al*., 2015; Primack *et al*., 2016) mention possible bias from incomplete adjustment.

Table 3 (see also Additional File 2 ( Lee, 2019)) gives illustrative examples of the effect of residual confounding. Situations 1 to 4 concern misclassification occurring in each direction, and correspond to situations 1, 2(a), 3(a), and 7(a) in Table 2, where no misclassification is assumed. Whereas in Table 2, the smoking predictor is assumed to be measured accurately, and adjustment for it would correct the observed gateway effect back to its true value (G _E_); this is not true when misclassification is present. Thus, in Situation 1, where G _E_ is set at 1, adjustment for the misclassified predictor does not fully correct for the confounding. Adjustment is useless where the misclassification rate is 50% with the observed gateway effect staying at its unadjusted value of 1.633. With a 10% misclassification rate, the value reduces to 1.281 so that almost half (281/633 = 44.4%) of the spurious increase in the OR remains. As misclassification rates reduce, the adjusted rate approaches its true value.

**Table 3.** Residual confounding due to misclassification of the smoking predictor.

Situation	Odds ratio relating vaping and predictor (K)	True OR of smoking for predictor (G _P_)	True RR gateway Effect (G _E_)	Observed gateway effect						
				Unadjusted	Adjusted for smoking predictor, with misclassification rate of					
					1%	2%	5%	10%	20%	50%
				**Misclassification is in both directions**						
1	5	4	1	1.633	1.034	1.067	1.157	1.281	1.455	1.633
2	2	4	1	1.233	1.013	1.026	1.060	1.106	1.169	1.233
3	5	2	1	1.266	1.015	1.029	1.068	1.120	1.193	1.266
4	5	4	2	3.149	2.058	2.115	2.270	2.486	2.802	3.149
				**Only false positives**						
5	5	4	1	1.633	1.030	1.058	1.136	1.246	1.408	1.625
				**Only false negatives**						
6	5	4	1	1.633	1.005	1.010	1.028	1.062	1.144	1.414

Notes: A misclassification rate of x% in both directions (considered in situations 1 to 4) implies that x% of those who have the factor that predicts smoking (e.g. risk takers) are wrongly classified as not having it, and x% of those who do not have the factor are wrongly classified as having it. Situations 5 and 6 consider misclassification in one direction. All models have P _1_ = P _2_ = 0.2 and P _A_ = 0.5.

The pattern is similar in Situations 2 and 3, where G _E_ remains at 1, but K and R _P_ are varied. Again, all of the bias remains with 50% misclassification, and almost half remains with 10% misclassification. This is also observed in Situation 4, where G _E_ = 2. Situations 5 and 6 are similar to Situation 1, except that misclassification is assumed to be unidirectional. The bias is similar to that observed in Situation 1, where only false positives occur (Situation 5), but is less where only false negatives are present (Situation 6). Thus poor specificity is more important than poor sensitivity as a source of bias.

### How might smoking prevalence be affected by any true effects of vaping?

It is important to understand how vaping might affect the prevalence of cigarette smoking. Not only might there be gateway effects, with vaping increasing the probability of subsequent smoking, but vaping by smokers might also increase their probability of smoking cessation. In an attempt to gain some understanding, we used the second sheet of the Excel program we developed (see also the mathematical details in Additional File 3 ( Lee, 2019)). The program considers 10,000 individuals, 2,000 in each of five strata that represent groups with varying smoking susceptibility. All individuals start as never users of either product and over time may switch to become current users of either product only, current dual users, or former users of either or both products (who currently use neither).

Where there is no effect of vaping on initiation or cessation, the user may vary the values of P _1_, R _1_, R _2_, R _3_ and R _4_ as defined in the methods section. These parameters are then used to update tobacco use status over time. Where there is an effect of vaping, the user may define G _1_, the gateway effect, and G _2_, the effect on the probability of smoking cessation.

Table 4 presents illustrative examples where G _1_ and G _2_ are set at 1.0. The five parameters P _1_, R _1_, R _2_, R _3_ and R _4_ are varied in turn, with the others held constant. Increasing e-cigarette initiation rates by increasing P _1_ (Block 1) reduces never-users, with a compensating increase in dual users and in the odds ratio relating current vaping to current smoking. In Block 1, it is assumed that there is no quitting or re-initiation (R _3_ = R _4_ = 0), the relative odds of initiation for successive strata (R _1_) are set at 5, and the initiation rates are assumed the same for both tobacco products (R _2_ = 1). Increasing R _1_ (Block 2), which increases the between stratum variation in smoking susceptibility, markedly increases the odds ratio relating current vaping to current smoking. The other Blocks keep P _1_ at 0.0002 and R _1_ at 5. Increasing R _2_ (Block 3) increases the frequency of smoking relative to vaping, the frequency of dual use and the odds ratio relating current vaping to current smoking. Increasing the quitting factor R _3_ (Block 4) reduces smokers, but the odds ratio is less affected. Furthermore, increasing the re-initiation factor R _4_ (Block 5) has little effect; in fact, the chance of someone initiating, quitting, then re-initiating during follow-up is small for the given parameter values.

**Table 4.** Effect of varying parameters on tobacco use and on the odds ratio relating vaping and smoking. All models assume G _1_ and G _2_ are 1.

Parameters						Percentage distribution of tobacco use					Odds ratio relating current vaping and smoking
Block	P _1_	R _1_	R _2_	R _3_	R _4_	Never use	E-cigarettes only	Cigarettes only	Dual use	Former use	
1	0.0001	5	1	0	0	88.0	5.3	5.3	1.4	0.0	4.52
	0.0002	5	1	0	0	80.6	7.6	7.6	4.3	0.0	5.93
	0.0005	5	1	0	0	69.3	9.1	9.1	12.5	0.0	10.44
	0.001	5	1	0	0	60.8	9.2	9.2	20.9	0.0	15.05
	0.002	5	1	0	0	52.1	9.3	9.3	29.4	0.0	17.85
2	0.0002	3	1	0	0	95.5	2.2	2.2	0.1	0.0	2.67
	0.0002	10	1	0	0	59.2	6.8	6.8	27.2	0.0	34.65
3	0.0002	5	0.25	0	0	85.6	10.5	2.3	1.3	0.0	4.83
	0.0002	5	0.5	0	0	83.9	9.4	4.3	2.5	0.0	5.19
	0.0002	5	2	0	0	75.7	5.3	12.5	6.5	0.0	7.43
	0.0002	5	4	0	0	69.5	3.2	18.7	8.7	0.0	10.15
4	0.0002	5	1	0.1	0	80.6	7.5	7.5	4.0	0.4	5.77
	0.0002	5	1	0.2	0	80.6	7.4	7.4	3.9	0.7	5.62
	0.0002	5	1	0.5	0	80.6	6.8	6.8	2.7	1.7	5.25
	0.0002	5	1	1.0	0	80.6	6.8	6.8	2.7	3.2	4.79
5	0.0002	5	1	0.2	0.1	80.6	7.4	7.4	3.9	0.7	5.61
	0.0002	5	1	0.2	0.2	80.6	7.4	7.4	3.9	0.7	5.60
	0.0002	5	1	0.2	0.5	80.6	7.4	7.5	3.9	0.6	5.58

P _1_, initiation rate of vaping in stratum 1; R _1_, relative odds of initiation for successive strata; R _2_, relative odds of initiation for smoking compared to vaping; R _3_, relative odds of quitting compared to initiation; R _4_, relative odds of re-initiation compared to initiation.

Table 5, Table 6 and Table 7 show how the relative odds of smoking are affected by variations, respectively, in G _1_ only, G _2_ only, or both. All these results set P _1_ = 0.0002, R _1_ = 5, and R _4_ = 0. With G _1_ and G _2_ set at 1.0, there are 11.28% cigarette smokers at the end of follow up: 7.43% smoking cigarettes only, and 3.85% dual users.

**Table 5.** Effect of varying G _1_ and R _2_ on smoking prevalence and the relative odds of smoking.

G _1_	R _2_	% cigarette smokers	Relative odds of smoking
1	1	11.28	1 (base)
2	1	12.31	1.10
3	1	13.03	1.18
4	1	13.56	1.23
5	1	13.93	1.28
1	0.25	3.56	1 (base)
5	0.25	5.23	1.50
1	0.5	6.56	1 (base)
5	0.5	8.93	1.40
1	2	17.37	1 (base)
5	2	19.66	1.16
1	2	23.22	1 (base)
5	4	24.79	1.09

G _1_, is the gateway effect; R _2_, the relative odds of initiation for smoking compared to vaping. The assumed values of the other parameters are P _1_ = 0.0002, R _1_ = 5, R _3_ = 0.2, R _4_ = 0, and G _2_ = 1. See text for the full definitions of these other parameters.

**Table 6.** Effect of varying G _2_, R _2_, and R _3_ on % smokers and relative smoking odds. Odds are expressed relative to the situation where G _1_ and G _2_ = 1.

G _2_	R _2_	R _3_	% smokers	Relative odds of smoking
1	1	0.2	11.28	1 (base)
2	1	0.2	11.16	0.99
3	1	0.2	11.06	0.98
4	1	0.2	10.96	0.97
5	1	0.2	10.87	0.96
1	1	0.5	11.28	1 (base)
5	1	0.5	9.82	0.93
1	0.25	0.2	3.56	1 (base)
5	0.25	0.2	3.52	0.99
1	0.5	0.2	6.56	1 (base)
5	0.5	0.2	6.42	0.98
1	2	0.2	17.37	1 (base)
5	2	0.2	16.40	0.93
1	4	0.2	23.22	1 (base)
5	4	0.2	21.55	0.91

G _2_ is the effect on the probability of smoking cessation. R _2_ is the relative odds of initiation for smoking compared to vaping. R _3_ is the relative odds of quitting compared to initiation. The assumed values of the other parameters are P _1_ = 0.0002, R _1_ = 5, R _4_ = 0, and G _1_ = 1. See text for the definitions of these other parameters.

**Table 7.** Odds of smoking according to variation in G _1_, G _2_, R _2_, and R _3_.

		G _1_	1	1	1	2	2	2	5	5	5
R _2_	R _3_	G _2_	1	2	5	1	2	5	1	2	5
0.25	0.2		1	0.997	0.989	1.143	1.140	1.129	1.497	1.491	1.473
0.25	0.5		1	0.993	0.975	1.136	1.128	1.104	1.473	1.459	1.421
0.5	0.2		1	0.994	0.978	1.129	1.121	1.101	1.398	1.387	1.358
0.5	0.5		1	0.987	0.955	1.123	1.107	1.066	1.383	1.359	1.301
1	0.2		1	0.989	0.960	1.104	1.091	1.056	1.276	1.259	1.217
1	0.5		1	0.976	0.926	1.102	1.073	1.012	1.272	1.236	1.160
2	0.2		1	0.979	0.933	1.072	1.050	0.999	1.164	1.140	1.086
2	0.5		1	0.961	0.897	1.073	1.031	0.959	1.169	1.124	1.046
4	0.2		1	0.967	0.908	1.043	1.011	0.952	1.090	1.059	1.002
4	0.5		1	0.952	0.890	1.048	0.999	0.936	1.103	1.055	0.990

G _1_ is the gateway effect. G _2_ is the effect on the probability of smoking cessation. R _2_ is the relative odds of initiation for smoking compared to vaping. R _3_ is the relative odds of quitting compared to initiation. The assumed values of the other parameters are P _1_ = 0.0002, R _1_ = 5, and R _4_ = 0. See text for the definitions of these other parameters.

As G _1_ increases to 5 ( Table 5), the percentage of cigarette smokers rises to 13.96%, giving an OR for smoking of 1.28 compared with G _1_ = 1. The increase in the percentage of smokers is proportionally much less than the increase in G _1_. Table 5 also shows that the gateway effect becomes more important as the relative initiation rate of cigarettes compared with that of e-cigarettes (R _2_) decreases.

As G _2_ increases from 1 to 5 ( Table 6), the percentage of smokers declines. The effect is less than for increasing G _1_, and it is in the opposite direction. This effect is increased as R _3_, the relative frequency of quitting to initiation, is increased. Table 6 also shows that G _2_ becomes more important as R _2_ increases.

In Table 7, both G _1_ and G _2_ are varied, with the relative odds of smoking shown for combinations of G _1_ and G _2_ = 1, 2, or 5 and for varying values of R _2_ and R _3_. The highest relative odds of smoking (1.497) are seen for the highest values of G _1_ and R _2_ and the lowest values of G _2_ and R _3_, while the lowest odds of smoking (0.890) are seen in the reverse situation.

The effects sometimes approximately cancel out. Given R _3_ < 1 (and it seems unlikely that quit rates would exceed initiation rates), effects of varying G _1_ tend to exceed effects of varying G _2_ where initiation rates are similar for vaping and smoking. However, where initiation rates for smoking are substantially higher (R _2_ = 4), effects of similar G _1_ and G _2_ values also approximately cancel out. Here, with relatively more smokers to quit, effects of varying G _2_ are more relevant.

While the above results are illustrative, one can derive five main general conclusions from them:

1. Large increases in G _1_, the gateway effect, result in proportionately much smaller increases in the smoking prevalence.
2. Gateway effects increase if initiation with vaping is more frequent than initiation with smoking.
3. Increasing G _2_ decreases smoking prevalence, but less than that from a similar increase in G _1_.
4. Effects of varying G _2_ increase as R _2_ and R _3_ increase, where there are more smokers who can quit.
5. With both G _1_ and G _2_ >1, the overall effect on smoking prevalence may be in either direction.

### Has introducing e-cigarettes affected smoking trends?

Smoking prevalence in U.S. youths had declined substantially even before vaping became popular. For example, the YRBS reported a decline in past 30-day smoking from 34.8% in 1995 to 15.7% in 2013. Has introducing e-cigarettes halted or even reversed this decline?

The NYTS is the only U.S. youth survey providing trend data on e-cigarette use over a reasonably long time period. In that survey, the prevalence of past 30-day vaping in each year from 2011–2015 was 1.0%, 2.0%, 2.9%, 9.2%, and 11.1%, respectively, for students in grades 6–12. In 2011–2013, vaping seems too rare to allow for assessment of any effect on smoking prevalence. To detect any effect, it seems more appropriate to compare the prevalence in 2014–2016 with that predicted from pre-existing trends.

Table 8 presents results from this comparison based on the U.S. NYTS, YRBS, MTF, and NSDUH surveys. All estimates are for past 30-day smoking. In all these surveys, smoking prevalence in 2014–2016 was less than predicted from the underlying trend, contrary to expectations of a definitive gateway effect being present. The tendency for the decline in prevalence to accelerate over 2014–2016 is evident regardless of sex or age. Additional File 4 ( Lee, 2019) provides further detail, including the observed data for the years before 2014, the fitted linear regression of log (p/(1 − p)) on year for years up to 2013, and graphical illustration that this linear regression model fits the data well.

**Table 8.** Observed smoking percentages in five studies compared to predictions from previous trends ^a^.

Study ^b^	Age/grade	Observed/predicted	2014	2015	2016
NYTS	6th–12th grade	Observed	6.2	6.1	-
		Predicted	8.22	7.77	-
MTF	8th grade	Observed	4.0	3.6	2.6
		Predicted	4.42	4.07	3.75
MTF	10th grade	Observed	7.2	6.3	4.9
		Predicted	9.56	9.03	8.53
MTF	12th grade	Observed	13.6	11.4	10.5
		Predicted	16.00	15.33	14.68
YRBS	9th–12th grade	Observed	-	11.8	-
	Males	Predicted	-	14.58	-
YRBS	9th–12th grade	Observed	-	9.7	-
	Females	Predicted	-	12.86	-
NSDUH	12–17 years	Observed	5.1	4.6	3.8
	Males	Predicted	5.29	4.68	4.14
NSDUH	12–17 years	Observed	4.6	3.8	3.1
	Females	Predicted	4.99	4.43	3.94
ONS	16–24 years	Observed	25.2	24.1	17.2
	Males	Predicted	23.0	22.7	22.4
ONS	16–24 years	Observed	20.9	22.9	16.0
	Females	Predicted	21.3	20.6	20.0

^a^Previous years used for estimation of trend: NYTS: 2004, 2006, 2009, 2011–2013; MTF, ONS: 2006, 2007, 2008, 2009, 2010, 2011, 2012, 2013; YRBS: 1995, 1997, 1999, 2001, 2003, 2005, 2007, 2009, 2011, 2013; NSDUH: 2009, 2010, 2011, 2012, 2013. ^b^MTF, Monitoring the Future; NSDUH, National Survey on Drug Use and Health; NYTS, National Youth Tobacco Survey; ONS, Office for National Statistics; YRBS, Youth Risk Behavior Survey.

The Smokefree Youth Survey in Great Britain also reported increasing vaping, with percentages of 4%, 6%, 11%, and 10% each year from 2013–2016, respectively, for 11–18-year-olds. As shown in Table 8, ONS data (for an older age group) also show no tendency for a rise in cigarette smoking given increasing vaping. Here, very small annual declines (about 0.3% in males and 0.7% in females) over 2006–2015 were followed by a much larger decline of 7% between 2015 and 2016.

These analyses, though clearly limited by the fact that we do not know what smoking prevalence would have been in the absence of e-cigarettes, do not suggest that introducing e-cigarettes has had any material adverse effect on smoking prevalence trends. If there were any gateway effect, it would be clearly outweighed by other issues, such as vaping providing an alternative to cigarettes for tobacco users, or changes in attitudes to smoking resulting from anti-tobacco prevention measures.

### Additional evidence on the likelihood of any marked gateway effect on smoking prevalence

Some additional evidence highlights the improbability of any substantial gateway effect of vaping on smoking prevalence. Based on the U.S. PATH study, Pearson *et al*. (2018) recently reported that only a small percentage of nonsmokers would be interested in using a hypothetical modified risk tobacco product. While these analyses were based on adults, it was possible to confirm this finding from the publicly available data files for 18–24-year-olds. Thus, the proportion “very or somewhat likely” to use such a product was much lower in those who had never used tobacco (57/1745 = 3.3%) than in those who had ever done so (2375/7282 = 32.7%). This difference was similar in both sexes. Those aged 12–17 years old were also asked in the PATH study if they had seen a tobacco product that claims to be safer than other tobacco products in the past 12 months and their likelihood of using such a product in the next 30 days. Again, the proportion answering “very or somewhat likely” was much lower in tobacco never-users (174/4673 = 3.72%) than in ever-users (264/1565 = 16.87%).

Based on the Canadian COMPASS study, Aleyan *et al*. (2018) compared rates of smoking initiation over a two-year follow-up period among a sample of 9,501 students between grades 9 and 11 (cigarette never-smokers), classified at baseline into four groups by current e-cigarette use and susceptibility to smoke and assessed using a three-item validated measure. Though the data in Figure 1 of that paper showed that in both unsusceptible and susceptible never-smokers, rates of smoking initiation were higher in current than noncurrent e-cigarette users, only an estimated 33/2646 = 1.2% of those who had tried smoking during the follow-up period were unsusceptible current e-cigarette users.

While evidence on the reported likelihood of future use of a new product or on susceptibility to smoking is less robust than that based on actual initiation of smoking, the results from these studies are consistent with the conclusion that even when there is some gateway effect, any resulting increase in smoking prevalence would be quite small.

### How might any true gateway effect modify the population health impact of introducing e-cigarettes?

It is possible for users of alternative tobacco products to have similar nicotine exposure to that of cigarette smokers but potentially much lower risk of smoking-related diseases (SRD). This is illustrated by the experience and prevalence of Swedish snuff (“snus”) use ( Agewall *et al*., 2002; Bolinder *et al*., 1997a; Bolinder *et al*., 1997b; Lee, 2011; Lee, 2013). As vaping presents significantly reduced exposure to toxicants and harmful and potentially harmful constituents compared with smoking ( National Academies of Sciences Engineering and Medicine, 2018), it would be expected that any harmful effects would be much lower. This hypothesis was subsequently endorsed by an expert group in 2014 ( Nutt *et al*., 2014).

This hypothesis is also further supported by various theoretical beneficial and adverse effects of vaping (see Table 9). The first major benefit of vaping (B1) concerns individuals who, in the absence of e-cigarettes, would have initiated smoking but instead take up vaping. They should have a much lower risk of SRDs than if they had smoked, given, for example, that the expert panel ( Nutt *et al*., 2014) estimated that relative to cigarettes, e-cigarettes likely pose a reduction in harm of up to 95%. This likely reduction in risk and harm also applies to benefit B2, where smokers who would otherwise have continued to smoke instead switch to vaping. While the benefits would take longer to emerge, as the adverse effects of past smoking need time to disappear, the reduction in risk associated with a switch to vaping should be a considerable proportion of that associated with quitting smoking. The health benefit would even be greater for established smokers, where vaping helps them quit (B3).

**Table 9.** Theoretical adverse and beneficial effects of e-cigarettes.

	Effect	Adverse or beneficial
A1	Vaping encourages initiation of smoking (gateway)	Adverse
A2	Smokers intending to quit switch to vaping instead	Adverse
A3	Smokers vape in addition to their normal cigarette consumption	Adverse
B1	Individuals who would otherwise have smoked vape instead	Beneficial
B2	Smokers who would have continued to smoke switch instead to vaping	Beneficial
B3	Vaping helps established smokers to quit	Beneficial
B4	Vaping helps established smokers to materially reduce cigarette consumption	Beneficial

Effects A3 and B4 in Table 9 both relate to those who switch from cigarettes to dual use of cigarettes and e-cigarettes. In theory, adverse effects might arise if smokers vape in addition to their usual cigarette consumption (A3), but this is probably implausible, because most dual users are likely to control their nicotine intake and actually reduce their cigarette consumption (B4), partially replacing cigarettes with e-cigarettes ( Berry *et al*., 2018; McNeill *et al*., 2014; McRobbie *et al*., 2014). Note that we use the word “materially” in the definition of effect B4 as theoretically, an adverse health impact might occur if a very small reduction in cigarettes is counterbalanced by a large increase in use of e-cigarettes.

While smokers intending to quit will do worse if they switch to e-cigarettes than if they had quit (A2), they will still do better than if they had continued to smoke.

Clearly, the greatest adverse effect arises for individuals who otherwise would not have smoked but are encouraged by vaping to do so (A1). However, considering the overall population health impact of this gateway effect, two points require emphasis. First, this is only likely to affect young people. Older people who decided not to start tobacco product use seem unlikely to start vaping, and even if they did so, it would probably be because they believe vaping to be much safer than the smoking they have already rejected ( Majeed *et al*., 2017; Popova *et al*., 2018; Xu *et al*., 2016; Zhu *et al*., 2013). Second, the arguments made earlier suggest that the number of new smokers originating from any true gateway-in effect should be quite low. Any adverse effects resulting from a gateway effect in young people (adverse effect A1) are likely to be outweighed in the general population by beneficial effect B1, as evidenced by the substantial numbers who have vaped but not smoked. The reduction in risk of SRDs for such individuals is likely to be almost as great as any increase resulting from a gateway effect.

## Discussion

Our analyses yield some major conclusions:

1. While the consistently increased adjusted gateway effect estimates appear compelling ( Table 1), they may be severely biased by ignoring established predictors of smoking initiation and residual confounding – a true causal effect remains to be demonstrated.
2. If a true gateway effect were to exist, it would probably have little effect on smoking prevalence.
3. No available evidence exists that increasing e-cigarette use has slowed the decline in smoking prevalence; indeed, the decline appears to have accelerated.

Though some publications have used the evidence for a gateway effect in youths to advocate regulations to curb vaping in the general population ( Miech *et al*., 2017; Primack *et al*., 2015; Soneji *et al*., 2017), others suggest that this over-interprets the evidence. Kozlowski & Warner (2017) point out the need “to better understand and assess confounding variables” and consider that the prospective studies merely “support that a minority of the relatively small number of e-cigarette triers—who haven’t also been experimenting with other tobacco products already—will go on to some experimentation with cigarettes.” Also, Levy *et al*. (2018) present calculations showing that “a strategy of replacing cigarette smoking with vaping would yield substantial life year gains, even under pessimistic assumptions [. . .].” Our argument that the gateway estimates are subject to relevant uncontrolled confounding is also supported by publications showing that vaping is associated with many factors associated with smoking ( Barrington-Trimis *et al*., 2015; Hanewinkel & Isensee, 2015; Temple *et al*., 2017). Unlike the implicit conclusion of the National Academies report ( National Academies of Sciences Engineering and Medicine, 2018), which found that control of confounding in the gateway-in studies has been adequate, our conclusions would suggest otherwise. Our conclusion is consistent with that of a recent publication ( Lee & Fry, 2019) which, based on data from the PATH study, found that making extensive adjustment for other risk factors, as well as limited adjustment for inaccuracy in some of these, explained as much as 87% of the observed gateway effect.

Our calculations indicating a lack of effect of e-cigarettes on smoking trends are further supported by Dutra & Glantz (2017), who concluded that “the introduction of e-cigarettes was not associated with a change in the linear decline in cigarette smoking among youth.” While studies ( McNeill *et al*., 2014) have argued against the likely population benefit of e-cigarettes due to their pervasive use among youths and young adults, as we have discussed, these analyses often fail to fully consider the limitations and weaknesses of the current evidence of the associated probabilities of e-cigarette use and cigarette smoking initiation in youths.

While various authors ( Hill & Camacho, 2017; Levy *et al*., 2018; Nutt *et al*., 2014) estimate that the introduction of e-cigarettes will have a beneficial population health impact, a recent publication Soneji *et al*. (2018) argues the opposite, presenting analyses estimating that “e-cigarette use in 2014 would lead to 1,510,000 years of life lost in the U.S. population.” The study reported that the large adverse effects from an estimated 168,000 cigarette never-smokers between 12 and 29 years old initiating cigarette smoking in 2015 and going on to become daily smokers would heavily outweigh the very small beneficial effects from an additional 2,070 current cigarette smoking adults aged 25–69 quitting smoking in 2015 and remaining abstinent for seven or more years.

These analyses have various weaknesses. First, they assume that there is no reduction in risk of SRDs for cigarette smokers who become dual users but do not quit cigarettes. Dual users are likely to reduce cigarette consumption ( Brose *et al*., 2015; Etter & Bullen, 2014; Farsalinos *et al*., 2016; Farsalinos *et al*., 2017; McRobbie *et al*., 2014) and hence their risk of SRD. Second, their analysis showing large adverse effects in youths relies heavily on the estimate of the gateway effect given by Soneji *et al*. (2017), which may largely result from confounding. Third, their analysis showing very small benefits from increased smoking quit rates in adults depended on an estimate derived from a prior review and meta-analysis by Kalkhoran & Glantz (2016) that estimated the OR of quitting among smokers with an interest in quitting. The estimate, and indeed the whole meta-analysis, has since drawn strong criticism from experts in the field for being inaccurate and misleading ( West *et al*., 2016).

Some limitations of our work and of the available evidence must be noted. First, many risk factors for smoking initiation are correlated, and had even more been adjusted for, this might not have significantly changed the adjusted effect estimates. Second, while we demonstrate that residual confounding may be important, we have not specifically reviewed evidence on how inaccurately particular risk factors are measured. However, nor has anyone else in this context, as far as we are aware. Third, we have not considered the likely persistence of any true gateway effects. Some youths who initiate tobacco use with vaping may try cigarettes but prefer vaping in the long run. Fourth, our consideration of the possible health effects of introducing e-cigarettes, was not based on rigorous modelling, but was somewhat speculative and based only on general considerations. Fifth, we did not consider evidence ( Doran *et al*., 2017; Leventhal *et al*., 2016) that vaping may be associated with heavier smoking, although this association may also be subject to confounding problems. Finally, our evidence on trends is based on data where the prevalence of vaping is little more than 10%. Where vaping is infrequent, any true gateway effect will only slightly increase smoking prevalence, as never smokers who have not vaped will far outweigh those who have. Any gateway effect will be greater if vaping is more common, so it is important to continue to monitor smoking and e-cigarette use trends to provide more conclusive insights.

## Conclusions

The existence of any true gateway effect has not been clearly demonstrated, due to limited control for confounding factors. Even if there is an effect, and subsequently, some individuals who otherwise would not have done so start to smoke cigarettes, any effect on smoking prevalence will probably be quite small, and general considerations suggest that any overall population health impact of introducing e-cigarettes is still likely be beneficial.

## Data availability

**Additional File 1. Confounding factors considered by studies of vaping as a possible gateway to smoking.** This .docx file lists those factors with published evidence of a relationship with smoking and gives those factors not considered in any of the 15 studies considered in Table 1.

**Additional File 2. Estimating the effects of omission of a confounding variable, or misclassification of it, on the association between vaping and subsequent initiation of smoking.** This .docx file presents details of the methodology used.

**Additional File 3. How might the prevalence of smoking be affected by any true gateway effects of vaping?** This .docx file presents details of the methodology used.

**Additional File 4. Comparing smoking prevalence in 2014–2016 with that predicted from preceding years.** For each of the 10 datasets considered in Table 8, this .xlsx file gives the model fitted to the prevalence in the years preceding 2014, as well as the observed and predicted prevalence, with graphs illustrating the appropriateness of the model and the tendency for the observed prevalence in 2014–2016 to be lower than predicted.

**Gateway calcs2. This .xlsx file is the Excel program used to produce the results presented in Additional Files 2 and 3.**

All data are available on OSF, DOI: https://doi.org/10.17605/OSF.IO/Z3ST5 ( Lee, 2019).

Data are available under the terms of the Creative Commons Zero "No rights reserved" data waiver (CC0 1.0 Public domain dedication).
