# Supplementary material for: Considerations related to vaping as a possible gateway into cigarette smoking: an analytical review
Source: F1000Res. 2019 Jul 22;7:1915. Originally published 2018 Dec 10. [Version 3] doi: 10.12688/f1000research.16928.3 (PMC6652100; doi:10.12688/f1000research.16928.3)
Supplement: Peer review history

# Peer review: Reviewer response for version 3

**DOI:** 10.5256/f1000research.21938.r51452

**Published:** 2019-07-22

**Authors:** Lion Shahab

**Affiliations:** 1 Department of Behavioural Science and Health, University College London (UCL), London, UK

The authors have addressed the previous comments.

I confirm that I have read this submission and believe that I have an appropriate level of expertise to confirm that it is of an acceptable scientific standard.

# Peer review: Reviewer response for version 2

**DOI:** 10.5256/f1000research.21197.r48811

**Published:** 2019-07-02

**Authors:** Lion Shahab

**Affiliations:** 1 Department of Behavioural Science and Health, University College London (UCL), London, UK

The authors have addressed most of the comments. I would still suggest to include some illustrative results in the abstract. Further, the fourth conclusion (”Finally, introducing e-cigarettes should lead to a reduced risk of SRD.”), as previously mentioned, is not based on primary data and has to be acknowledged to be speculative at this stage, without using more rigorous modelling (e.g. see papers by Levy *et al.* or the models included in the NASEM report) to quantify this statement.

I confirm that I have read this submission and believe that I have an appropriate level of expertise to confirm that it is of an acceptable scientific standard, however I have significant reservations, as outlined above.

# Response

**Published:** 2019-07-17

**Authors:** Peter Lee

**Affiliations:** P.N.Lee Statistics and Computing Ltd, UK

We thank Dr Shahab for his further comments. As a result we have extensively rewritten the abstract to include some of the illustrative results he required, though the abstract limit of 300 words precluded us giving very much detail.

We have also made a number of changes to the paper to make the limitations of our work clearer, and to improve the English in one or two places. We have removed the conclusion 'Finally, introducing e-cigarettes should lead to a reduced risk of SRD' from the four main conclusions listed at the beginning of the discussion, and made it clearer that this conclusion is based on general considerations rather than rigorous modelling.

# Peer review: Reviewer response for version 2

**DOI:** 10.5256/f1000research.21197.r48812

**Published:** 2019-06-13

**Authors:** Sandra I. Sulsky

**Affiliations:** 1 Ramboll US Corporation, Amherst, MA, USA

The revised paper addresses prior comments. Overall, this work demonstrates the importance of considering the totality of evidence when a public health relevant question is the topic of debate. Its quantitative evaluation of the potential effects of uncontrolled confounding on risk estimation are useful for any research setting (i.e., not just tobacco-related or harm reduction research).

I confirm that I have read this submission and believe that I have an appropriate level of expertise to confirm that it is of an acceptable scientific standard.

# Peer review: Reviewer response for version 1

**DOI:** 10.5256/f1000research.18509.r45925

**Published:** 2019-04-12

**Authors:** Sandra I. Sulsky

**Affiliations:** 1 Ramboll US Corporation, Amherst, MA, USA

This ambitious paper by Lee and colleagues seems to have several aims, and I fear that the paper suffers from including them all, rather than separating them out into two or more focused manuscripts.

 The authors provide a review of the literature discussing what is currently known about the association between use of combusted and electronic tobacco products, they evaluate the likelihood of electronic products providing a gateway in to smoking based on seemingly informal, qualitative trend analyses, and they provide an important discussion about the potential for residual or otherwise uncontrolled confounding to explain or partially explain associations that have been noted in the literature. They also provide data from a series of simulations. The simulations demonstrate the interdependence among various tobacco use patterns in predicting population harm or benefit resulting from electronic tobacco products.

 The most important points from the literature-based sections of the paper are a) the difference between predicted and observed smoking prevalence over time, which should reduce the fear that a large gateway to smoking effect is currently operating; b) the likelihood that misclassification and uncontrolled confounding play a role in the results reported by others, and probably lead to an overestimate of gateway-in effect.

 The key findings from the simulations are a bit harder to uncover, due to a lack of detail in the methods section and some glossing over of assumptions. The authors found that a gateway-in effect is unlikely to be large enough to substantially affect population health. This is based on the assumptions that a) the current situation, in which smoking is far more prevalent than electronic cigarette use, will remain stable in at least the short run; b) the current higher likelihood of initiating tobacco use with cigarettes vs electronic products will remain stable in at least the short run; and c) the assumptions that the risks associated with electronic products are understood and are definitely lower than risks associated with smoking. If any of these input assumptions were varied, the simulation results would probably be different, and possibly much different. Some of the authors' strongest statements are due to differences between relative and absolute effects: Small percentages of a large underlying population (smokers) affect a large number of people, and large percentages of a small underlying population (electronic product users or initiators) affect a small number of people. 

 Apart from these conceptual concerns, it would strengthen the paper if the input into the simulations were better defined and justified.

 The analyses presented in Table 8 seem to be the most informative of the simulations. Estimating the rate of the combination of smoking OR vaping OR dual use and comparing the prevalence in each year to the prevalence predicted by trends in smoking would give clues about the population level effects of e-products. Has overall use increased or stayed the same?

I confirm that I have read this submission and believe that I have an appropriate level of expertise to confirm that it is of an acceptable scientific standard, however I have significant reservations, as outlined above.

# Response

**Published:** 2019-05-13

**Authors:** Peter Lee

**Affiliations:** P.N.Lee Statistics and Computing Ltd, UK

Dr Sulsky makes three main points:

The paper may be better as multiple focussed manuscripts. We did not attempt this, our main purpose being to cover a range of issues relevant to vaping. Note that two of us also recently published in F1000Research on “Investigating gateway effects using the PATH study”.

Some key findings from the simulations are hard to uncover and some assumptions not clearly stated. We made numerous amendments to answer these points.

It would also be useful to study trends in combined prevalence of vaping and/or smoking. As the paper’s interest concerns effects of vaping on smoking prevalence, and as interpretation of upward trends in combined use is problematic (they may well reduce risk) we did not change the paper here.

# Peer review: Reviewer response for version 1

**DOI:** 10.5256/f1000research.18509.r45926

**Published:** 2019-03-28

**Authors:** Lion Shahab

**Affiliations:** 1 Department of Behavioural Science and Health, University College London (UCL), London, UK

This is an interesting and valuable contribution to the field on the hotly debated topic of whether or not e-cigarettes function as a gateway into subsequent combustible cigarette use. Of particular value here is the analysis of the impact of residual confounding on creating spurious associations in observational studies. However, there are a number of issues in the other analyses I would recommend addressing.

  

1. Abstract: This is currently rather data free. I would suggest including some illustrative results here.
2. Results: Particularly in relation to Tables 5-7, I wonder if it would be possible to condense results somewhat (or to move some of these results into supplementary files). It seems that it is not strictly necessary to include all modelled effects, especially since not all are discussed. This makes this section at times difficult to follow. A better way to structure the Tables might be to link these explicitly to the 5 conclusions presented on pages 9 and 10.
3. Terminology: I am not sure that the term “gateway-out” is helpful. At the very least, it would not represent the only way to construe this concept insofar as it could be argued that in a hypothetical scenario the opposite to the “gateway-in” might be the avoidance of uptake of smoking in the first place (another “gateway-out” not related to smoking cessation where adolescents who would have smoked try an e-cigarette and never start smoking). It might therefore be helpful to clarify the difference between those gateway effects away from smoking
4. Change in smoking trends: I am not entirely convinced by this analysis for a number of reasons. First, very little information is provided about the actual analysis undertaken. I would expect to see fitting parameters for various functions to model trends in smoking prevalence in the period prior to 2014. Are these assumed to be linear, logarithmic, polynomial etc.? More detail is required here. A more appropriate way to analyse the data would be to conduct segmented regression or timeseries analysis. Second, based on the current analysis, the problem (somewhat acknowledged on top of page 11) is that it might still be the case that e-cigarettes functioned as a “gateway in” since we do not know what smoking prevalence would have been in the absence of e-cigarettes (and may have been even lower). I don’t think this is likely but this issue should be acknowledged more explicitly, perhaps making it clear that such arguments would require “special pleading”, i.e. one would need to postulate additional environmental changes which could explain a continued decrease in smoking prevalence even in the presence of a gateway effect.
5. Second paragraph on page 11 does not seem to add anything (it’s not clear how these cognitive measures in PATH relate to actual behaviour and so it’s quite weak evidence). Suggest dropping this.
6. Table 9: The counterpart to A3 is not presented, i.e. dual use which results in reduction of combustible cigarettes. If there is a commensurate reduction in cigarette consumption associated with an increase in e-cigarette use (if dual users reduce cigarette consumption sufficiently) there may be beneficial health effects (so perhaps this effect should be added). In fact, perhaps a more nuanced discussion of dual use is needed on page 12, given that it may have both positive and negative effects and encompasses such a wide range of behavioural patterns. Please note that the statement on page 11 “The adverse effect for smokers switching to vaping instead of quitting smoking (A2) is not ideal but would still confer some benefits, given what is known regarding the reduced harm and toxicity of e-cigarettes" is logically inconsistent. If a smoker intending to quit starts using e-cigarettes instead of quitting, there is no benefit (as e-cigarette use even if less harmful than smoking does not confer any benefit over stopping completely).
7. The discussion on end of page 11 and top of page 12 feels rather qualitative. Either provide more quantitative analysis of modelled impact on health effects or move this to the discussion. You don’t really provide any primary data to support your conclusion ”Finally, introducing e-cigarettes should lead to a reduced risk of SRD.” This is rather speculative at this stage without more rigorous modelling as was done e.g. in the NASEM report.

I confirm that I have read this submission and believe that I have an appropriate level of expertise to confirm that it is of an acceptable scientific standard, however I have significant reservations, as outlined above.

# Response

**Published:** 2019-05-13

**Authors:** Peter Lee

**Affiliations:** P.N.Lee Statistics and Computing Ltd, UK

Taking Dr Shahab’s numbered points:

1. As the paper covers numerous issues and the conclusions are intended to be general, and as abstract length is limited, we left the abstract unaltered.
2. We prefer not to change Tables 5 and 7. They are short and reducing them makes it harder to see effects of variation in the different parameters.
3. We have abandoned the terms gateway-in and gateway-out. Gateway-in is now called the gateway effect, and gateway-out is explained more generally.
4. A new additional file (4) gives fuller detail. The model used – linear in log (p/(1-p)) - was actually described originally, the additional information suggesting this is a reasonable approach. The discussion has been increased here.
5. Rather than dropping some material in the section “Additional evidence on …”, we prefer adding a paragraph clarifying the data limitations.
6. Table 9 and the related discussion have been amended along the lines suggested.
7. We moved the last two paragraphs of results to the discussion.
